# The Incidence and Impact of Atrial Fibrillation on Hospitalized Coronavirus Disease‐2019 Patients

**DOI:** 10.1002/clc.24320

**Published:** 2024-07-19

**Authors:** Haiming Niu, Jianwei Li, Catherine Teng, Xiaojia Lu, Chengyue Jin, Peng Cai, Ao Shi, Xiaoqing Shen, Qiqi Chen, Miaolian Chen, Yong Yuan, Pengyang Li

**Affiliations:** ^1^ Department of Critical Care Medicine Zhongshan People's Hospital Zhongshan China; ^2^ Department of Medicine, Division of Cardiology University of Texas Health Science Center San Antonio Texas USA; ^3^ Department of Cardiology Mount Sinai Beth Israel, Icahn School of Medicine at Mount Sinai New York New York USA; ^4^ Department of Cardiology The First Affiliated Hospital of Shantou University Medical College Shantou China; ^5^ Department of Mathematical Sciences Worcester Polytechnic Institute Worcester Massachusetts USA; ^6^ Faculty of Medicine St. George University of London London UK; ^7^ Department of Cardiovasculogy Zhongshan People's Hospital Zhongshan China; ^8^ Division of Cardiology, Pauley Heart Center Virginia Commonwealth University Richmond Virginia USA

Dear Editor,

Thank you for your insightful inquiries regarding our study on the clinical implications of atrial fibrillation (AF) in patients with COVID‐19. We appreciate the opportunity to further discuss and clarify our findings and methodologies.

## 1 Inflammatory Status and AF as an Indicator of COVID‐19 Severity

Our observations reveal that the cohort with COVID‐19 and AF demonstrated increased mortality and a higher incidence of major cardiovascular and cerebral complications. This pattern suggests that AF might reflect the severity of COVID‐19. However, as noted in our limitations, the National Inpatient Sample (NIS) lacks specific laboratory data, such as ESR and CRP levels, which restricts our capacity to compare the inflammatory status between patients with and without AF directly. While our study underscores the association of AF with poorer in‐hospital outcomes in hospitalized COVID‐19 patients, it does not establish causality. We concur with your perspective that the presence of AF could signify a more severe inflammatory status of COVID‐19.

## 2 Comparison With AF Alone

The comparison between patients hospitalized primarily for COVID‐19 with AF and those with AF as the primary condition presents intriguing possibilities for understanding the interplay between AF and COVID‐19. However, our study design and data source posed significant challenges for such a comparison. The NIS database categorizes conditions based on primary and secondary diagnoses without distinguishing between new onset and pre‐existing AF. This limitation complicates the differentiation between patients hospitalized due to the severity of AF itself and those for whom AF is a comorbid condition alongside COVID‐19.

Moreover, conceptualizing a comparison group of “AF alone” encounters methodological hurdles. The group could potentially consist of patients hospitalized with AF as the primary diagnosis, which often indicates acute episodes requiring immediate intervention, or patients with AF as a secondary diagnosis hospitalized for various other reasons. The clinical scenarios and patient profiles in these groups are markedly diverse, making direct comparisons with our COVID‐19 and AF cohort challenging. The acute presentation of AF in the former contrasts with the broader spectrum of AF severity in COVID‐19 patients, while the latter's diverse hospitalization reasons introduce confounding variables that hinder a clean comparison.

Given these considerations, our analysis focused on elucidating the association between AF and COVID‐19 outcomes within the limitations of available data. While we acknowledge the potential value that such a comparison might offer, technical and methodological constraints led us to focus our study scope on patients hospitalized with COVID‐19, with and without AF as a comorbid condition.

## 3 Cardiac Arrest and Ventricular Arrhythmias

In our study, we identified them using ICD‐10 codes for ventricular fibrillation (VF) and flutter (I490) and ventricular tachycardia (VT) (I472), excluding premature ventricular contractions (see Supplement table). The relationship between COVID‐19, AF, and cardiac arrest involves multifaceted mechanisms. These include the potential for AF in COVID‐19 patients to degenerate into life‐threatening ventricular arrhythmias such as VF and VT [1, 2]. Factors contributing to these severe outcomes may include acute myocardial injury, electrolyte imbalances, and the systemic inflammatory response associated with COVID‐19, which could exacerbate underlying cardiovascular conditions [3, 4]. Our analysis, constrained by the scope of data available in the NIS, highlights the need for further studies with detailed clinical data to explore these pathways comprehensively.

We hope that this response offers a clearer understanding of our study's findings and the complexities involved in analyzing the interplay between AF and COVID‐19. We appreciate the dialogue that your inquiries have sparked and look forward to further discussions on this vital topic.
